# Correction: Novel decorated aluminium(iii) phthalocyanine complex with the application of MWCNTs on electrodes: electrochemical non-enzymatic oxidation and reduction of glucose and hydrogen peroxide

**DOI:** 10.1039/d6ra90039a

**Published:** 2026-04-09

**Authors:** Mounesh, P. Manikanta, K. R. Venugopala Reddy, Manickam Selvaraj, C. C. Vidyasagar, Bhari Mallanna Nagaraja

**Affiliations:** a Centre for Nano and Material Science (CNMS), Jain (Deemed-to-be University) Jain Global Campus, Kanakapura Bangalore Karnataka 562112 India mounesh.m.nayak@gmail.com bm.nagaraja@jainuniversity.ac.in; b Department of Studies and Research in Chemistry Vijayanagara Sri Krishnadevaraya University Ballari – 583105 Karnataka India; c Department of Chemistry, Faculty of Science, King Khalid University PO Box 9004 Abha 61413 Saudi Arabia; d Research Centre for Advanced Materials Science (RCAMS), King Khalid University PO Box 9004 Abha 61413 Saudi Arabia; e Department of Studies and Research in Chemistry, Rani Channamma University Belagavi – 591156 Karnataka India

## Abstract

Correction for ‘Novel decorated aluminium(iii) phthalocyanine complex with the application of MWCNTs on electrodes: electrochemical non-enzymatic oxidation and reduction of glucose and hydrogen peroxide’ by Mounesh *et al.*, *RSC Adv.*, 2023, **13**, 20723–20736, https://doi.org/10.1039/D3RA02617E.

The authors regret that incorrect versions of Fig. S1 and S3 were provided. The SI of the original article has been updated to include the correct versions of Fig. S1 and S3, which have been assessed by an independent expert. The corrected SI figures are shown below, in addition to changes to the discussion of these figures in the main text of the original article.
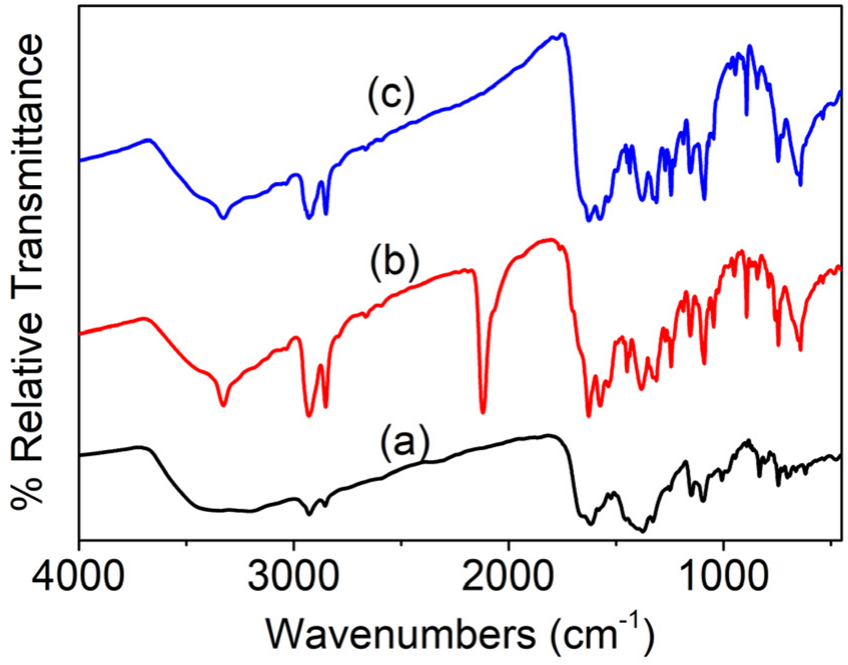


**Fig. S1:** FTIR spectra of (a) Al(iii)TCAPc, (b) Al(iii)TMQNCAPc, and (c) Al(iii)TMQNCAPc@MWCNTs.
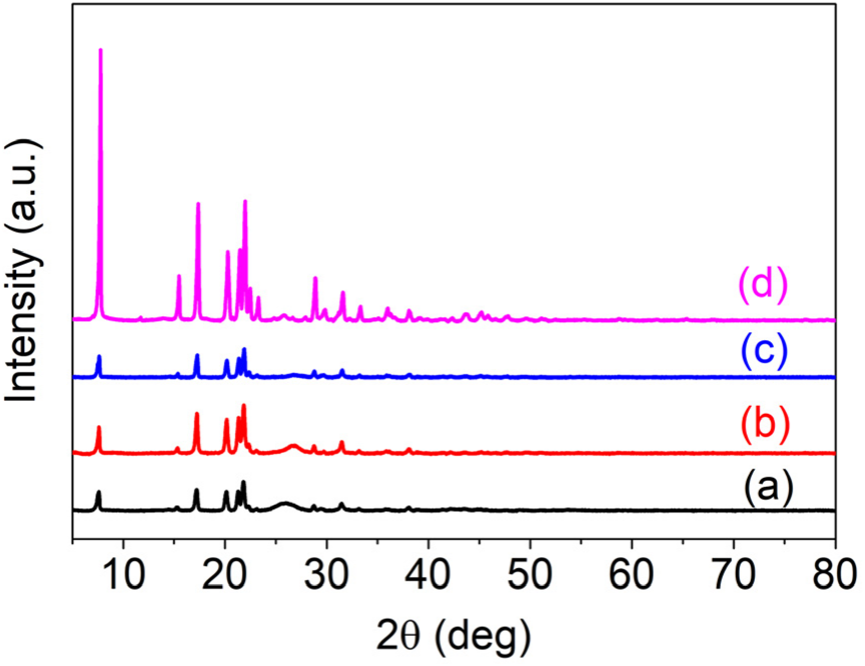


**Fig. S3:** Powder XRD analysis of (a) Al(iii)TCAPc, (b) Al(iii)TMQNCAPc, (c) MWCNTs and (d) Al(iii)TMQNCAPc@MWCNTs.

On pages 20725–20726, the text should read:

“All the synthetic aluminum phthalocyanine complexes had FTIR spectra with peaks in the range of 500 to 4000 cm^−1^. The FTIR spectra of compounds (a), (b) and (c) are shown in Fig. S1, where all the compounds show vibrational bands at 1509–1689 cm^−1^, which corresponds to the C=C stretch, and at 2967–2809 cm^−1^, corresponding to the aromatic C–H stretch. In the region of 3639–3097 cm^−1^ (–OH and –NH_2_), as shown in Fig. S1(a), the carboxylic acid group of Al(iii)TCAPc appeared at 3639–3097 cm^−1^ but Fig. S1(b) shows the disappearance of the –COOH group and the appearance of a new peak for the substituted amide group (Al(iii)TMQNCAPc) at 3333 cm^−1^ (–CONH). Fig. S1(c) shows the spectrum of Al(iii)TMQNCAPc@MWCNTs, where a peak appears in the region of 2984–2815 cm^−1^ (Ar–CH), vibrations caused by the stretching of the C=N and C=C groups appear at around 1678–1492 cm^−1^, and a sharp peak in the region of 1464–1351 cm^−1^ corresponds to C=O. Additionally, the bands at 1317, 1272, 1238, 1154, 1075, 889, 832, 743, 643, and 534 cm^−1^ correspond to the vibrational bands that support the presence of functional groups in the Al(iii)TMQNCAPc ring.”

On page 20727, the text should read:

“The powder X-ray diffraction study of Al(iii)TCAPc, Al(iii) TMQNCAPc, MWCNTs and Al(iii)TMQNCAPc@MWCNTs was performed in the 2*θ* range of 5–80°, and the results are shown in Fig. S3 as offset curves for (a) Al(iii)TCAPc, (b) Al(iii)TMQNCAPc, (c) MWCNTs and (d) Al(iii)TMQNCAPc@MWCNTs. The crystal structure and size of the QDs were clarified by PXRD investigation. The replacement complex and parent PCs displayed the same patterns. However, compared to similar aluminum PCs, the patterns exhibited a higher peak intensity throughout the complex. The crystallinity was described using powder X-ray diffraction patterns.^32,33^ The diffraction pattern of Al(iii)TMQNCAPc shows sharp peaks at 7°, 15°, 17°, 20°, 22°, 28°, 31°, 33°, 36°, 38°, and 45° with slightly lower intensity, which was caused by the conjugation of the π-electrons, manipulating its stacking arrangement. The shapes of the X-ray diffraction patterns indicate that Al(iii)TCAPc, Al(iii)TMQNCAPc, MWCNTs and Al(iii)TMQNCAPc@MWCNTs were crystalline in nature.^34,35^”

The Royal Society of Chemistry apologises for these errors and any consequent inconvenience to authors and readers.

